# *Arabidopsis* CPK5 Phosphorylates the Chitin Receptor LYK5 to Regulate Plant Innate Immunity

**DOI:** 10.3389/fpls.2020.00702

**Published:** 2020-06-11

**Authors:** Congcong Huang, Yijia Yan, Huilin Zhao, Ying Ye, Yangrong Cao

**Affiliations:** State Key Laboratory of Agricultural Microbiology, College of Life Science and Technology, Huazhong Agricultural University, Wuhan, China

**Keywords:** chitin receptor, plant defense, PTI, CPK5, LYK5

## Abstract

Chitin, a major component of the fungal cell wall, triggers plant innate immunity in *Arabidopsis via* a receptor complex including two major lysin motif receptor-like kinases, AtLYK5, and AtCERK1. Although AtLYK5 has been proposed to be a major chitin-binding receptor, the pseudokinase domain of AtLYK5 is required to mediate chitin-triggered immune responses in plants. In this study, 48 AtLYK5-interacting proteins were identified using immunoprecipitation and mass spectrometry assay. Among them, *Arabidopsis* CALCIUM-DEPENDENT PROTEIN KINASE 5 (AtCPK5) is a protein kinase interacting with both AtLYK5 and AtCERK1. Chitin-induced immune responses are inhibited in both *Arabidopsis atcpk5* and *atcpk5/6* mutant plants. AtLYK5 and AtLYK4 but not AtCERK1 are phosphorylated by AtCPK5 and AtCPK6 *in vitro*. Liquid chromatography-tandem mass spectrometry (LC-MS/MS) analysis and *in vitro* kinase assay identified that Ser-323 and Ser-542 of AtLYK5 are important phosphorylation residues by AtCPK5. Transgenic *Arabidopsis* expressing either AtLYK5-S323A or AtLYK5-S542A in the *atlyk5-2* mutant only partially rescue the defects in chitin-triggered MPK3/MPK6 phosphorylation. Overexpression of AtCPK5 could increase AtCERK1 protein level after chitin treatment. These data proposed a model in which AtCPK5 directly phosphorylates AtLYK5 and regulates chitin-induced defense responses in *Arabidopsis*.

## Introduction

Plants have evolved complicated immune responses to detect and defend against pathogens. Plants employ pattern recognition receptors (PRRs) to perceive conserved microbe-derived molecules and to initiate the first layer of immune response (Jones and Dangl, 2006). This response is characterized by the generation of reactive oxygen species (ROS), the activation of mitogen-activated protein kinase (MAPK) cascades, defense-related gene expression and Ca^2+^ influx, to fight against pathogens’ attack (Dangl, 2013; Li B. et al., 2016; Li L. et al., 2016). Pattern-trigged immunity (PTI) in response to microbe-associated molecular patterns (MAMPs) represents the first layer of plant immunity. However, successful pathogens inject effectors into host cells to target PRRs or the downstream components involved in the PTI pathway, to attenuate or block the host’s immune response (Ma et al., 2015, 2017; Diepold et al., 2017; Henry et al., 2017). However, plants possess a set of resistance genes, which encode nucleotide-binding domain and leucine-rich repeat intracellular receptors, to directly or indirectly detect the presence of the effector and initiate effector-triggered immunity (ETI) (Jones and Dangl, 2006; Jacob et al., 2013; Cui et al., 2015).

One of the best-characterized PRRs is *Arabidopsis* FLAGELLIN SENSITIVE2 (AtFLS2), a leucine rich-repeat-containing receptor kinase that perceives flg22 (Gomez-Gomez and Boller, 2000). Following perception of flg22, another LRR-receptor-like kinase, AtBAK1, is recruited to form receptor complex with AtFLS2, and then phosphorylation events occur between AtFLS2/AtBAK1 and BIK1, a receptor-like cytoplasmic kinase induced by *Botrytis* infection, to initiate the early immune response (Chinchilla et al., 2007; Heese et al., 2007; Lu et al., 2010; Lin et al., 2014). In addition, AtBAK1 functions as a universal co-receptor for multiple PRRs, including AtCERK1 to mediate MAMP-triggered immunity (Li et al., 2002; Chinchilla et al., 2007; Postma et al., 2016; Gong et al., 2019).

Chitin is the major component of fungal cell walls and a typical MAMP that causes PTI defense response (Wan et al., 2004). The first identified chitin receptor is chitin elicitor-binding protein in rice (OsCEBiP), a lysin motif-containing protein (Kaku et al., 2006). Following chitin perception, OsCEBiP is induced to form receptor complex with rice CHITIN ELICTOR RECEPTOR KINASE 1 (OsCERK1), another chitin receptor that contains a lysin motif within the ectodomain and an intracellular kinase domain (Hayafune et al., 2014). In *Arabidopsis*, AtLYK5 is proposed to play a major role in mediating chitin perception. The known knowledge suggests that a sandwich-type model (with chitin sandwiched between AtCERK1 and AtLYK5 or between OsCERK1 and OsCEBiP) explains the formation of the chitin receptor complex, because chitin can induce complex formation between AtCERK1 and AtLYK5 (Cao et al., 2014; Hayafune et al., 2014). AtLYK5 could also be phosphorylated by the AtCERK1 kinase domain *in vitro* (Erwig et al., 2017), suggesting that the kinase domain of AtLYK5 is important for mediating chitin signaling in plants.

It remains unknown how the two chitin receptors function cooperatively, as well as other components function in chitin-induced defense signal transduction and regulation. Changes in protein levels might be one way for AtLYK5 to regulate the immune response, which is similar to that of AtFLS2 after flg22 elicitation (Robatzek et al., 2006; Lu et al., 2011; Cui et al., 2018). The protein level of AtLYK5 is regulated by an E3 ligase (Liao et al., 2017). Before chitin treatment, AtLYK5 interacts with AtPUB13, a U-box-containing E3 ligase, which might mediate the proteasomal degradation of AtLYK5. However, chitin induced AtPUB13 disassociates from AtLYK5, which results in AtLYK5 accumulation in addition to endocytosis (Erwig et al., 2017; Liao et al., 2017). AtCERK1 is another chitin receptor kinase whose Y428 residue is crucial for transduction of the defense signal. However, mutation at Y428 does not affect the *in vitro* kinase activity of AtCERK1 (Liu et al., 2018). Mutation of residue Y428 also abolishes cell death caused transient expression of AtCERK1 in *Nicotiana benthamiana* (Suzuki et al., 2018). However, mutation of residues T479 and T573 of AtCERK1, two phosphorylation sites located within the kinase domain, abolishes kinase activity and expression of several downstream defense-related genes, which implies the existence of a complicated regulation on different phosphorylation residues of AtCERK1 in chitin-induced defense response (Suzuki et al., 2016).

It is of great interest to identify the essential downstream components in chitin-induced defense pathway. It was reported that the AtPBL27, a receptor-like cytoplasmic kinase, is directly regulated by AtCERK1 and connects chitin perception to the activation of the MAPK cascade (Shinya et al., 2014; Yamada et al., 2016). However, other studies reported that members from RLCK VII-4, but not AtPBL27, play central roles in chitin-induced defense pathway and links the chitin receptor to MAPK cascade activation (Bi et al., 2018; Rao et al., 2018). Such contradiction might be due to different plant growing conditions or chitin preparations (Gong et al., 2020).

Whereas, it remained unknown whether there are some other components in addition to CERK1, regulate LYK5 cytoplasmic domain to mediate chitin induced immune response. Here, we performed mass spectrometry analysis to identify the substrates of the chitin receptor after affinity purification of AtLYK5, and demonstrated that AtCPK5 interacts with both AtCERK1 and AtLYK5. Both the *atcpk5* and *atcpk5/6* null mutants exhibited deficiencies in chitin-induced defense response compared to wild type *Arabidopsis*, which demonstrates that AtCPK5 is a component of the chitin-induced defense pathway. AtLYK5 and AtLYK4 are phosphorylated by AtCPK5 and AtCPK6 *in vitro*, however, no *trans-*phosphorylations were observed between AtCPK5 and AtCERK1 in *in vitro* kinase assay. The phosphorylation sites Ser323 and Ser542 of AtLYK5 was identified as targets of AtCPK5. These data uncover a new mechanism of AtCPK5 in chitin-induced defense responses in plants.

## Materials and Methods

### Plant Materials and Growth Condition

The *Arabidopsis thaliana* plants used in the study include *atcerk1-2* (GABI-KAT 096F09) (Miya et al., 2007), *atlyk5-2* (SALK_131911C) (Cao et al., 2014), *atcpk5* (SAIL_657C06), *atcpk6* (SALK_025460C) (Boudsocq et al., 2010), and wild-type Col-0. All *Arabidopsis* plants were grown at the temperature 22°C under a condition of 16 h light/8 h dark cycle with 70% humidity. Generally, 4-week-old *Arabidopsis* plants grown in soil or 10-day-old seedlings grown in plates containing 1/2 Murashige and Skoog (MS) with 0.5% agar and 1% sucrose were used for chitin induced physiological analyses.

### Gene Cloning and Plasmids Construct

The coding sequences of both *AtCPK5* and *AtCPK6* were amplified and introduced into the entry vector pDONR207. After Gateway LR reaction, *AtCPK5* and *AtCPK6* were cloned into destination binary vectors pGWB511 and pGWB-n/cYFP (modified from pGWB514, in which the n/cYFP were inserted behind HA-tag). The generated binary vectors were electroporated into *Agrobacterium* for either stable transformation in *Arabidopsis* or bimolecular fluorescence complementation assays in *N. benthamiana*. For kinase assay, the coding sequences of *LYK5-CD* (301-664aa) and *LYK4-CD* (295-612 aa) were cloned into pGEX-5X-1 between *Eco*RI and *Eco*RI, CERK1-CD, AtCPK5, and AtCPK6 were cloned into pMAL-C2X-1 between *Eco*RI and *Sal*I. The residues, D441 of CERK1, D221 of CPK5, and D209 of CPK6 were substituted into valine or alanine using PCR-based site-directed mutagenesis method.

### Stable Transformation and Transient Expression

The pGWB511-CPK5 construct was introduced into *Agrobacterium tumefaciens* strain GV3101 using electroporation. The agrobacterial culture was pelleted at 2000 g for 5 min and resuspended in 5% sucrose supplemented with 0.02% Silwet L-77. The floral dip method was applied to generate transgenic *Arabidopsis* either in wild type Col-0 or in the *atlyk5* null mutant plant expressing AtLYK5-HA under its native promoter (Cao et al., 2014). For transient expression, *A*. *tumefaciens* strain EHA105 was resuspended in a buffer containing 10 mM MES-KOH buffer, 10 mM MgCl_2_ and 0.15 μM acetosyringone (PH = 5.7) with an OD600 value at 1.0. Fully expanded leaves from 4-week-old *N. benthamiana* were used for infiltration with *Agrobacterium* using a needleless syringe.

### *In vitro* Kinase Assay

Either GST- or MBP-Tag fused recombinant proteins were expressed and purified from *Escherichia coli* strain BL21 (DE3). In brief, culture of *E. coli* strain harboring plasmid was added with IPTG (0.3 mM) at OD_600_ ∼ 0.6 for protein expression at 28°C for 5 h. PBS buffer was used for purification of both GST- and MBP-tag fused recombinant proteins. Protein purification of the GST (Genscript, Nanjing, China) and MBP (New England Biolabs, Ipswich, MA, United States) tags was performed according to the manufacturer’s instructions. For the *in vitro* kinase assay, about 0.5 μg protein were incubated in the kinase buffer (10 mM MgCl_2_, 1 mM MnCl_2_, 1 mM DTT, 50 mM Tris–HCl, PH 7.5, 5 μCi γ-^[32]^P ATP) for 30 min at 24°C. After that, the reaction was terminated after adding SDS loading buffer followed by separation on 10% SDS-PAGE gel. Autoradiography was performed using a phosphor screen and a phosphorimager. All the phosphorylation assays were repeated at least twice.

### Co-immunoprecipitation Assay

Plant tissues either from *Arabidopsis* seedings or *N. benthamiana* for Co-IP assay were ground in a lysis buffer (50 mM Tris–HCl PH 7.5, 150 mM NaCl, 2 mM EDTA, 1% Triton X-100, 10% glycerol (v/v), 1× complete protease inhibitors) on ice for 30 min before centrifuging at 13000 rpm at 4°C for 30 min. The supernatant of each sample was added with 20 μl anti-FLAG agarose (Sigma, St Louis, MO, United States) followed by end-to-end rotation at 4°C for 3 h. About 1 ml lysis buffer was used to wash agarose bead at least five times. The Co-IP assay was performed according to the methods described by Liao et al. (2017).

### MAPK Phosphorylation, ROS Production, Callose Deposition

For the MAPK phosphorylation assay, 10-day-old *Arabidopsis* seedings were treated with chitin (25 μg/ml) for 5 min and 15 min. Crude proteins were lysed in the lysis buffer followed by separation on 10% SDS-PAGE gel. The phosphorylated MPK3/6 was detected using anti-P44/P42 antibody (Cell Signaling Technology, Frankfurt, Germany). The process of western blot was performed by Cao et al. (2014). For ROS production, leave disks were punched from 4-week-old *Arabidopsis* leaves and treated with chitin (25 μg/ml) or H_2_O and determined using a plate reader as described by Cao et al. (Liao et al., 2017). For callose deposition assay, 4-week old *Arabidopsis* leaves were infiltered with chitin (25 μg/ml) or flg22 (500 nM) for 24 h. Leaves were stained with aniline blue and visualized under UV light as described by Cao et al. (2013).

### RNA Extract and qRT-PCR

The total RNA was extracted using the Ultrapure RNA Kit from the *Arabidopsis* seedings treated with chitin (CWBIO, Wuhan, China). Reverse transcription was performed using M-MLV reverse transcriptase (Promega, Madison, WI, United States) as the protocol described. QPCR was performed as described by Cao et al. (2013) with three biological repeats. All the primers used in qRT-PCR analysis are listed in the primer list.

## Results

### AtCPK5 Associates With AtLYK5

Our previous work showed that the hetero-receptor complex consisting of AtCERK1 and AtLYK5 and its homolog AtLYK4 is required for chitin recognition and signal transduction in plants. In the chitin receptor model in *Arabidopsis*, the major role of AtLYK5 was proposed to bind to chitin, whereas AtCERK1 is involved in signal transduction due to its active kinase domain. However, the role of the pseudokinase domain of AtLYK5 remains largely unknown. To address this, the AtLYK5-Myc protein complex was immunoprecipitated from AtLYK5-Myc transgenic plants using anti-Myc antibody and analyzed using mass spectrometry. Based on the analyzed peptides, 48 proteins were identified as candidates of AtLYK5-interacting proteins (Supplementary Table S1).

**FIGURE 1:**
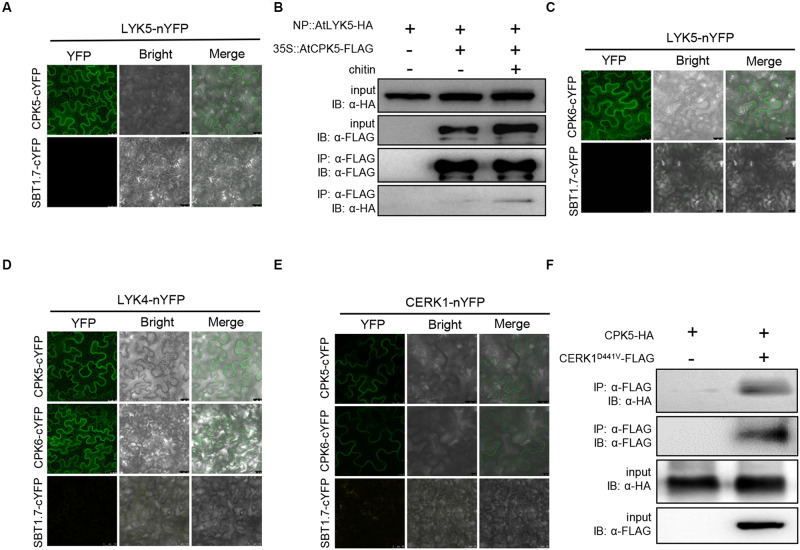
AtCPK5 and AtCPK6 interact with chitin receptors in *Arabidopsis*. **(A,C–E)** AtCPK5 and AtCPK6 interact with chitin receptors with the BiFC assay. The AtCPK5 and AtCPK6 are fused to the C-terminus of YFP, the chitin receptors are fused to the N-terminus of YPF. The corresponding EHA105 strains carrying the target plasmid are transiently expressed in the *N. benthamiana* and observed with a confocal laser-scanning microscope. The gene SBT1.7 is served as negative control. Bars were shown in each picture. **(B)** Co-IP assay between AtCPK5 and AtLYK5 with or without chitin treatment in *Arabidopsis*. AtCPK5 fused 3XFLAG tag was introduced to AtLYK5-HA under its native promoter background, 15-day-old seedlings from liquid MS medium were harvested and treated with 25 μg/mL chitin (+) or water (–) for 15 min. Co-immunoprecipitation was made using anti-FLAG and anti-HA antibody. The assay was carried out for twice and displayed same result. **(F)** Co-IP assay between AtCPK5 and AtCERK1 in *N. benthamiana.* AtCERK1 fused 3XFLAG tag and AtCPK5 fused 3XHA tag at the end of each coding sequence. The two genes were coexpressed in Nicotiana leaves. Co-immunoprecipitation was performed using anti-FLAG and anti-HA antibodies. The assay was carried out twice with same result.

Among the identified proteins, AtCPK5 and AtCPK6 were studied in this report. The interaction between AtLYK5 and AtCPK5 was validated using bimolecular fluorescence complementation (BiFC) assay. AtLYK5-nYFP and AtCPK5-cYFP or the negative control AtSBT1.7-cYFP were transiently expressed under control of the 35S promoter in *N. benthamiana* leaves. Strong fluorescence signal was detected in the cells expressing both AtCPK5-cYFP and AtLYK5-nYFP compared with negative control (Figure 1A), indicating that AtCPK5 interact with AtLYK5. To confirm the interaction between AtLYK5 and AtCPK5, 3 × FLAG-tagged AtCPK5 was transgenically expressed in the *atlyk5-2* mutant plants rescued by AtLYK5-HA under its native promoter. AtLYK5-HA was coimmunoprecipitated with anti-FLAG antibody in the sample before chitin treatment (Figure 1B). However, much stronger band representing the coimmunoprecipitated AtLYK5-HA was detected in the plant tissue 30 min post chitin treatment, indicating that the interaction between AtLYK5 and AtCPK5 could be enhanced by chitin treatment.

Because AtCPK6 and AtLYK4 are the closest homologs of AtCPK5 and AtLYK5, respectively, we then examined the interactions between these proteins. Plant cells expressing both AtLYK5-nYFP and AtCPK6-cYFP showed strong fluorescence signals compared with negative controls (Figure 1C). We then examined AtLYK4-nYFP and AtCPK5-cYFP, AtLYK4-nYFP and AtCPK6-cYFP interactions using BiFC in *N. benthamiana* leaves. As shown in Figure 1D, strong fluorescence signals were detected to indicate that AtLYK4 interacts with both AtCPK5 and AtCPK6. Then we tested interaction between AtCERK1 and AtCPK5 and AtCPK6 using BiFC assay. AtCERK1-nYFP and AtCPK5-cYFP, AtCERK1-nYFP and AtCPK6-cYFP were coexpressed, respectively, in *N. benthamiana* leaves with fluorescence signals detected compared with the negative controls (Figure 1E). To confirm the interaction between AtCPK5 and AtCERK1, AtCPK5-HA and AtCERK1^D441V^-FLAG were transiently coexpressed in *N. benthamiana* leaves. After coimmunoprecipitating with anti-FLAG antibody, the band representing AtCPK5-HA was detected using anti-HA antibody (Figure 1F). Taken together, these data indicate that both AtCPK5 and AtCPK6 interact with AtCERK1 and AtLYK5.

### Both AtCPK5 and AtCPK6 Are Required for Chitin-Induced Immune Response in *Arabidopsis*

**FIGURE 2:**
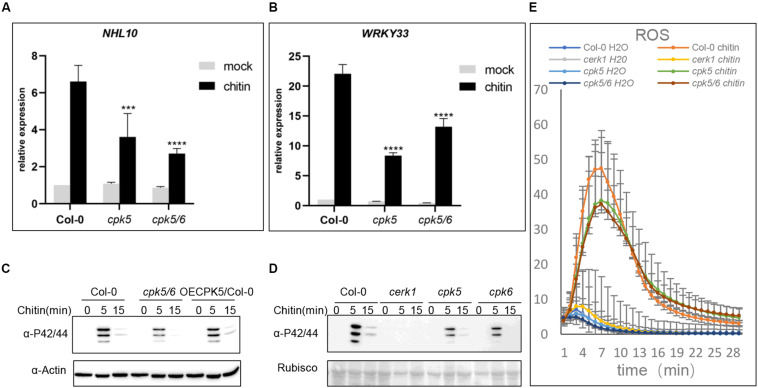
The *atcpk5* null mutant shows defective defense response after chitin elicitation. **(A,B)** Chitin-induced *AtNHL10* **(A)** and *AtWRKY33* **(B)** expression was analyzed using qRT-PCR in the wild type, *atcerk1*, *atcpk5, atcpk5/6* mutant plants were treated with 25 μg/mL chitin for 15 min. ACTIN2 was used as internal standard. Data are presented as mean ± SD, *P* < 0.001 (*n* = 3, one-way ANOVA, Tukey post-test, three independent experiments). **(C)** Chitin-induced MAPK activation in *atcpk5/6*, *35S::CPK5* in wild type background and **(D)** *atcerk1*, *atcpk5, atcpk6* mutants compared with the Col-0 wild type. 10-day-old seedlings were treated with 25 μg/mL chitin or H_2_O. All samples were harvested at the indicated times for immunoblot analysis with anti-P44/P42 antibody. **(E)** Accumulation of reactive oxygen species (ROS) in 4-week-old plants from Col-0 wild-type, *atcpk5, atcerk1, atcpk5/6* mutant plants. ROS was measured 30 min after treatment with 25 μg/mL chitin, or water using luminol; RLU, relative light units. Error bar represent ± SD of the mean. ***P < 0.005, ****P < 0.001.

The observations above showed that AtCPK5 and AtCPK6 interact with AtLYK5. We then hypothesized that AtCPK5 and AtCPK6 are involved in chitin-triggered immune responses in *Arabidopsis*. To test this, chitin-induced physiological changes were examined in the *atcpk5* mutants, *atcpk5/6* double mutants and wild type *Arabidopsis*. 10-day-old *Arabidopsis* seedlings were treated with chitin to analyze the expression of defense-related genes *AtNHL10* and *AtWRKY33*. Expressions of *AtNHL10* and *AtWRKY33* were detected at much lower levels in the *atcpk5* and *atcpk5/6* than that in wild type plants treated with chitin, indicating that both AtCPK5 and AtCPK6 are required for chitin-induced immune response in *Arabidopsis* (Figures 2A,B).

Because MAPK cascades are essential for defense signal transduction, we examined MAPK3/6 phosphorylation in the *atcpk5*, *atcpk6*, and *atcpk5/6* mutant plants. The degree of MAPK3/6 phosphorylation was detected at lower levels in all the mutant plants than that in either AtCPK5 overexpressing plants or wild type plants (Figures 2C,D). AtCPK5 could phosphorylates AtRBOHD to activate ROS generation (Dubiella et al., 2013), therefore, we examined ROS production in wild type, *atcpk5*, and *atcpk5/6* mutants in response to chitin treatment. We observed decreased ROS generation in both the *atcpk5* and *atcpk5/6* mutant plants compared to wild type (Figure 2E). Callose deposition induced by chitin treatment but not by flg22 treatment was significantly lower in both the *atcpk5* and *atcpk5/6* than that in wild type (Supplementary Figure S1A). We hypothesized that the kinase activity of AtCPK5 is activated after chitin treatment, which is similar to flg22 treatment. To do this, *CPK5-FLAG* was transgenically expressed in wild-type *Arabidopsis* (Supplementary Figure S1B). AtCPK5 was able to respond to chitin, similar to flg22. However, no difference in band molecular weight was observed in plants treated with chitin compared with the mock treatment (Supplementary Figure S1C). These data clearly indicate that AtCPK5 is involved in the chitin-induced defense response.

### AtLYK5 and AtLYK4 but Not AtCERK1 Are Phosphorylated by AtCPK5 and AtCPK6 *in vitro*

**FIGURE 3:**
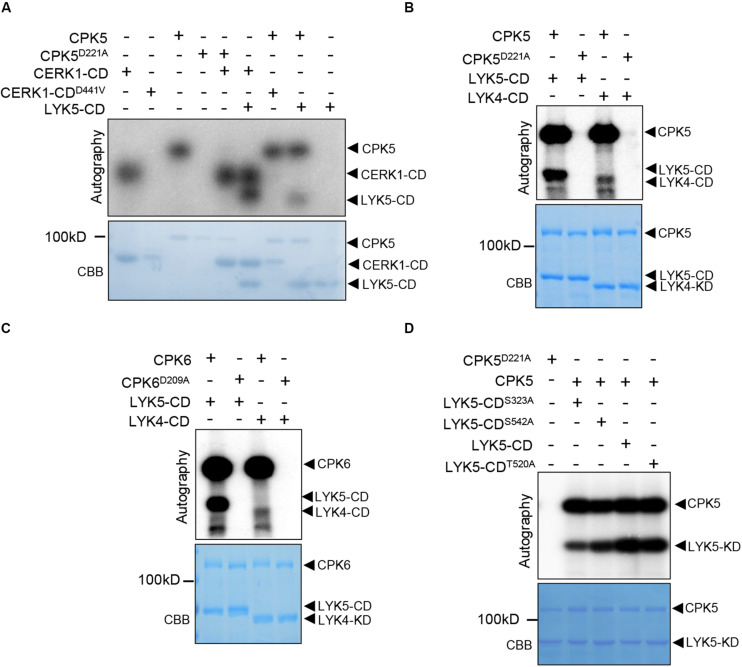
The AtLYK5 cytoplasmic domain but not that of AtCERK1 is phosphorylated by AtCPK5. *In vitro* kinase assays were measured in presence of γ−−^[32^^]^P-ATP. In each figure, phosphorylated proteins were visualized using autoradiography (top panel). SDS-PAGE gel stained with Coomassie brilliant blue (bottom panel). **(A)** AtCPK5 phosphorylates the cytoplasmic domain of AtLYK5. CPK5^D221A^, AtCPK5 kinase dead variant. AtCERK1-CD^D441V^, AtCERK1-CD kinase dead variant. **(B)** AtCPK5 phosphorylates the cytoplasmic domain of AtLYK5 and AtLYK4. **(C)** AtCPK6 phosphorylates the cytoplasmic domain of AtLYK5 and AtLYK4. **(D)** AtCPK5 phosphorylates the cytoplasmic domain of AtLYK5^S323A^, AtLYK5^S542A,^ AtLYK5^T520A^, and AtLYK5.

Above data indicated that both AtCPK5 and AtCPK6 are important for chitin-triggered immunity in plants. Therefore, we then asked how AtCPK5 regulates chitin response in *Arabidopsis*. Because both AtLYK5, AtCERK1 and AtCPK5 are protein kinases, we performed *in vitro* kinase assays between AtCPK5 and chitin receptors to test whether AtCPK5 could phosphorylates AtCERK1 and AtLYK5 or *vice versa*. To do this, AtCPK5, AtCPK6 and AtCERK1-CD (cytoplasmic domain) were fused to the MBP (maltose binding protein), while LYK5-CD and LYK4-CD were fused to the GST-tag and expressed in *E. coli*. AtCPK5^*D221A*^, AtCPK6^*D209A*^, and AtCERK1^*D441V*^-CD were used as negative controls due to the mutation at the key residue (Asp) at the activation loop which is required for catalyzation of kinase reaction. In the sample containing CERK1^*D441V*^-CD and MBP-CPK5, no phosphorylated signal of CERK1^*D441V*^-CD was detected in the autoradiography. Similarly, no phosphorylated signal of MBP-CPK5^*D221A*^ was detected when incubated with MBP-CERK1-CD (Figure 3A), indicating that no *trans-*phosphorylation occurred between CERK1 and CPK5. However, we observed an autoradiography band of GST-LYK5-CD when it was incubated with MBP-CPK5, in a similar way to the phosphorylation of AtLYK5-CD by AtCERK1-CD. These data indicate that in addition to AtCERK1, AtCPK5 is another protein kinase that could directly phosphorylate AtLYK5.

Because AtLYK5 and AtLYK4 are close homologs, we performed kinase assay between AtCPK5 and AtLYK4-CD and demonstrated that AtLYK4-CD was also phosphorylated by AtCPK5 (Figure 3B). AtCPK6 protein is a highly homologous protein of AtCPK5 and also involved in plant defense, therefore, we performed *in vitro* kinase assay between AtCPK6 and AtLYK5-CD as well as with AtLYK4-CD. We found that both GST-LYK5-CD and GST-LYK4-CD could be phosphorylated by AtCPK6 (Figure 3C). These data clearly indicate that AtCPK5 and AtCPK6 phosphorylate the cytoplasmic domain of AtLYK5 and AtLYK4, but not CERK1.

We queried the potential mechanism by which AtLYK5 is regulated by AtCPK5. After *in vitro* phosphorylation assay, the phosphorylated GST-LYK5-CD was analyzed using liquid chromatography-tandem mass spectrometry (LC-MS/vMS) analysis. The phosphopeptides containing either Ser323 or Ser542 of AtLYK5 contributed to more than 75 percent of the total phosphopeptides detected using LC-MS/vMS (Supplementary Figures S2A,B). Sequence homology analysis indicated that Ser323 of AtLYK5 is analogous to Ser314 of AtLYK4 (Supplementary Figure 2C). We then substituted all of the residues with alanine and fused the resulting protein with the C-terminus of GST. We reperformed *in vitro* kinase assay after purifying these proteins from *E. coli* and observed that mutation at these residues except T520, a phosphorylation site as a control, resulted in significant decrease in the intensity of the phosphorylated LYK5-CD band (Figure 3D), indicating that AtCPK5 phosphorylates AtLYK5 at S323 and S542. Sequence alignment showed that Ser542 of AtLYK5 is located within a loop of the kinase domain (Supplementary Figure 2C). All these data suggest that both S323 and S542 might be important for the function of AtLYK5.

**FIGURE 4:**
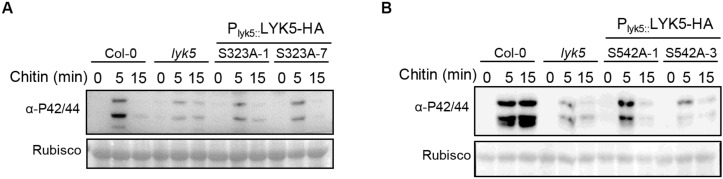
The Ser323 and Ser542 residues of LYK5 are required for chitin induced MAPK3/6 phosphorylation. **(A)** and **(B)**, MAPK3/6 phosphorylation were detected using immunoblot in LYK5^*S323A*^ and LYK5^*S542A*^ background as well as wild type Col-0, *atlyk5* was used as negative control. 10-day-old seeds were grown in 1/2 MS plates containing 1% sucrose and treated with chitin (25 μg/ml) at indicated time.

In order to clarify the role of Ser323 and Ser542 of AtLYK5 in chitin-induced immunity, we substituted the two phospho-sites into Alanine using PCR-based site directed mutagenesis method, respectively. AtLYK5^*S323A*^ and AtLYK5^*S542A*^ were fused HA at C-terminate and transformed into *atlyk5-2* null mutant background under its native promoter (Supplementary Figures 3A,B). MPK3/6 phosphorylation was measured in these transgenic seedlings following chitin treatment compared with wild-type *Arabidopsis*. As shown in Figures 4A,B, it turned out that weak phosphorylated bands of MAPK3/6 occurred in the transgenic plants expressing LYK5^*S323A*^ or LYK5^*S542A*^ compared to Col-0, whereas, LYK5-HA can rescue the diminished MAPK3/6 phosphorylation in *atlyk5-2* mutant plant (Supplementary Figure 3C). These data indicate that the residues of Ser323 and Ser542 are required for AtLYK5 to mediate the chitin-induced MAPK cascade activation.

### Overexpression of AtCPK5 Increases AtCERK1 Protein Level

**FIGURE 5:**
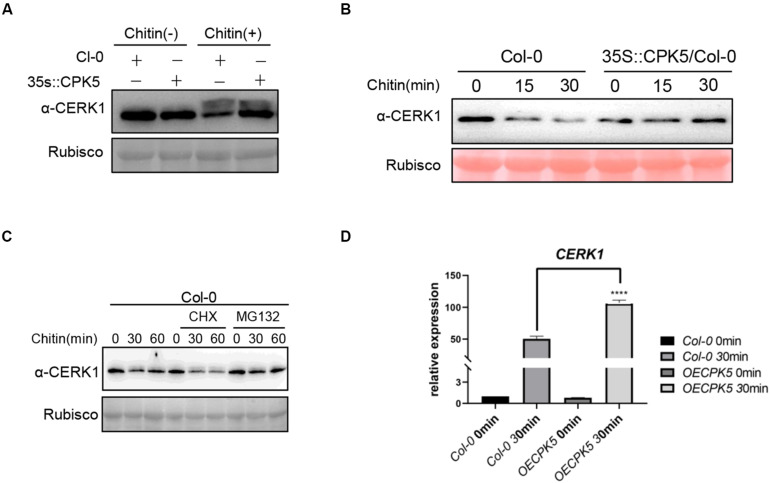
AtCPK5 stables AtCERK1 protein level after chitin treatment. **(A)** AtCERK1 accumulates in the AtCPK5 overexpressing background. Col-0 and AtCPK5 overexpressing seedling were grown on plates containing 1/2 MS for 10 days. Treated these seedlings with chitin (25 μg/ml) for 30 min, then performed immunoblot using the α-CERK1 antibody. **(B)** AtCPK5 stables AtCERK1 protein level after chitin treatment. Col-0 and AtCPK5 overexpressing seedlings were grown on plates containing 1/2 MS for 10 days. Then treated these seedlings using chitin (25 μg/ml) for indicated time and subjected to perform immunoblot using α-CERK1 antibody. **(C)** AtCERK1 undergoes degradation induced by chitin in Col-0 wildtype with or without CHX or MG132 treatment. 10-day-old seedlings from Col-0 were treated with chitin, chitin and CHX, or chitin and MG132 for indicated time. Total protein was subjected to SDS-PAGE and detected by immunoblot with α-CERK1 antibody. **(D)** AtCPK5 regulated *AtCERK1* transcript after chitin treatment. QRT-PCR analysis *AtCERK1* expression in the wild type and AtCPK5 overexpressing seedings after chitin elicitation for 30 min. All the sample were grown on ½ MS plate for 10 days and then these seedlings were treated with chitin (25 μg/mL) and ACTIN2 was used as internal standard. Data are presented as mean ± SD, *P* < 0.001 (*n* = 3, one-way ANOVA, Tukey post-test, three independent experiments). *****P < 0.001.

Above data showed that AtCPK5 could interact with AtCERK1 and AtLYK5. However, phosphorylation event was only detected between AtCPK5 and AtLYK5 but not between AtCPK5 and AtCERK1. We wonder how AtCPK5 is involved in regulating chitin-triggered immune response in plants. Chitin treatment could induce the phosphorylation of AtCERK1 which could be easily observed as a band retardation on SDS-PAGE gel. We perform immunoblot assay to detect AtCERK1 protein in the AtCPK5-overexpressing *Arabidopsis* after chitin treatment using anti-CERK1 antibody. We found that the band shift representing phosphorylated CERK1 after chitin treatment in both wild type Col-0 and AtCPK5-overexpressing plants (Figure 5A), indicating that overexpression of CPK5 might not alter the phosphorylation of CERK1 after chitin treatment. Chitin treatment could decrease the protein level of AtCERK1 in *Arabidopsis*. We found the protein level of AtCERK1 is much higher in AtCPK5-overexpressing plants compared with wild type *Arabidopsis* after chitin treatment (Figure 5A), suggesting that AtCPK5 might suppress the degradation of AtCERK1 after chitin treatment. To confirm the results, we treated wild type *Arabidopsis* and AtCPK5 overexpressing *Arabidopsis* seedlings with chitin. AtCPK5 overexpressing *Arabidopsis* showed increased AtCERK1 protein level after chitin treatment (Figure 5B). Cycloheximide (CHX), an inhibitor for protein synthesis in eukaryotic cells, and MG132, a chemical inhibiting protein degradation *via* 26S proteasome, were used to examine the mechanisms of suppression of AtCERK1 degradation by AtCPK5 after chitin treatment. We found that treatment with CHX could significantly reduce the protein level of AtCERK1 compared with chitin treatment along (Figure 5C). While treatment with MG132 could significantly increase protein level of AtCERK1 after chitin treatment (Figure 5C). These data indicate that chitin treatment could induce the degradation of AtCERK1 *via* 26S proteasome, while AtCPK5 could stabilize AtCERK1 protein level. Because overexpression of AtCPK5 could increase protein level of AtCERK1. We then examined whether AtCPK5 could regulate the transcriptional level of AtCERK1. The transcriptional levels of *AtCERK1* in the AtCPK5-overexpressing plants were detected at much higher levels than that in wild type Col-0 plants (Figure 5D). The data indicate that AtCPK5 has a role in regulating the protein levels of AtCERK1.

## Discussion

Perception of chitin by AtLYK5 and AtCERK1 to activate the defense response represents the first layer of the immune response in *Arabidopsis* (Miya et al., 2007; Liu et al., 2012). AtLYK5 is another major chitin receptor to form receptor complex with AtCERK1 and to be phosphorylated by AtCERK1 (Cao et al., 2014; Erwig et al., 2017). Our data add a new clue that AtCPK5 and AtCPK6 act as new components interacting with AtLYK5 and AtCERK1 to regulate chitin-induced immunity response in plants. Our data proposed a possible mode in which AtCPK5 and AtCPK6 directly phosphorylate AtLYK5 and stabilize AtCERK1 to fine tune chitin-triggered immunity in plants.

The role of AtCPK5 and AtCPK6 have been shown to regulate plant innate immunity. One direct evidence from the finding is that AtCPK5 and AtCPK6 could target RBOHD, a key enzyme required for ROS production in response to MAMP treatment. How AtCPK5 and AtCPK6 are activated is unclear. The current study demonstrated that AtCPK5 and AtCPK6 regulate chitin-triggered immunity in *Arabidopsis* through direct association with AtLYK5. As protein kinase, AtCPK5 and AtCPK6 could phosphorylate AtLYK5, as AtCERK1 does. Although AtCPK5 forms protein complex with both AtCERK1 and AtLYK5, there is no phosphorylation events between AtCERK1 and AtCPK5. The direct roles of AtCPK5 and AtCPK6 are to phosphorylate AtLYK5 at Ser323 and Ser542 to positively regulate chitin-triggered immunity. Even though, it was already known that AtCERK1 could directly phosphorylate AtLYK5. Thus, whether AtCERK1 shares the same phosphorylation sites with AtCPK5 is of a great interest to be tested.

Recent publication showed that BAK1, the universal co-receptor for most PRRs, directly associates and phosphorylates AtCEKR1 at the juxtamembrane domain, such phosphorylation protect CERK1 from degradation (Gong et al., 2019). The interesting finding in this study is that AtCPK5 and AtCPK6 could associate with AtCERK1, but no phosphorylation events could be detected. The possible role of AtCPK5 might stabilize the protein of AtCERK1 after chitin treatment. AtCPK5 is located at the plasma membrane through N-myristoylation (Lu and Hrabak, 2013). The remaining question is whether BAK1 associates with AtCPK5, whereas, need to be determined.

Previous research has indicated that AtCPK5 responds to bacterial AvrRpt2 to mediate signal transduction from plasma membrane to the nucleus to phosphorylate WRKY transcriptional factors (Gao et al., 2013). Activated AtCPK5 phosphorylated RBOHD and mediates ROS production in response to flg22 (Dubiella et al., 2013). These results clearly demonstrate that AtCPK5 possesses universal functions in plant immunity. The *atcpk5* and *atcpk5/6* mutant plants showed defects in response to chitin treatment support the above conclusion that AtCPK5 is a universal player in response to multiple stimuli. In addition to directly phosphorylate AtLYK4 and AtLYK5, AtCPK5 could stabilize protein level of AtCERK1 in response to chitin treatment. The possible role of AtCPK5 is to increase transcriptional level of AtCERK1 in *Arabidopsis* post chitin treatment. Due to the truth that AtCPK5 could associate with WRKY transcription factor to directly regulate the expression of target genes (Gao et al., 2013). Therefore, it is possible that some transcription factors which are directly targeted by AtCPK5 to regulate the expression of AtCERK1. Overall, our data provide sufficient data showing that AtCPK5 and AtCPK6 work as essential components in the chitin-triggered immune response in *Arabidopsis*.

## Data Availability Statement

All datasets generated for this study are included in the article/Supplementary Material.

## Author Contributions

CH and YC conceived the idea and designed the experiments, provided the proposal for the research, analyzed the data, and wrote the manuscript. CH, YYa, HZ, and YYe performed the experiments.

## Conflict of Interest

The authors declare that the research was conducted in the absence of any commercial or financial relationships that could be construed as a potential conflict of interest.
