# Supplementary material for: Arabidopsis CPK5 Phosphorylates the Chitin Receptor LYK5 to Regulate Plant Innate Immunity
Source: Front Plant Sci. 2020 Jun 11;11:702. doi: 10.3389/fpls.2020.00702 (PMC7300259; doi:10.3389/fpls.2020.00702)
Supplement: Supplementary file 1 [file Data_Sheet_1.docx]

Supplemental Table S1. Identified proteins from AtLYK5 receptor complex.

| Protein IDs | Gene names | Peptide counts (all) | Score |
| --- | --- | --- | --- |
| P0DH96; P0DH95 | CAM4; CAM1 | 9;9 | 323.31 |
| P17562 | SAM2 | 28 | 323.31 |
| O65639 | CSP1 | 20 | 323.31 |
| Q42479; Q3E9C0; Q9FMP5 | CPK3 | 31;1;1 | 323.31 |
| Q9FI56; Q8VYJ7 | CLPC1 | 56;1 | 323.31 |
| Q9SI75 | CPEFG | 22 | 249.51 |
| Q0WPH8 | NEK5 | 26 | 198.81 |
| O48963 | PHOT1 | 20 | 191.86 |
| F4J6F6; F4HYG2; F4HPN2 | IREH1 | 31;3;1 | 145.61 |
| O22193; Q5XEZ8 | PUB4 | 18;1 | 135.68 |
| Q9XF89 | LHCB5 | 11 | 93.965 |
| P48347; Q9S9Z8 | GRF10 | 15;3 | 86.296 |
| Q8GYN5 | RIN4 | 6 | 85.713 |
| Q9SIV2 | RPN1A | 21 | 81.206 |
| Q9SHE8 | PSAF | 7 | 78.437 |
| Q8LPJ4; Q9LID6 | ABCE2 | 15;1 | 73.731 |
| Q9LTX9 | HSP70-7 | 27 | 68.619 |
| O22808 | LYK5 | 7 | 65.107 |
| P49107 | PSAN | 10 | 62.987 |
| P48349; Q9C5W6 | GRF6 | 14;1 | 62.623 |
| Q9C5T4; Q9SK33 | WRKY18 | 12;1 | 60.96 |
| Q8LDW9 | XTH9 | 4 | 16.761 |
| Q9LSP8 | CMTA6 | 2 | 12.327 |
| Q93VB2 | ATG18A | 6 | 12.213 |
| Q9FFI2 | ATG5 | 5 | 11.113 |
| Q4FE47 | XBAT35 | 1 | 8.2287 |
| Q38872; Q9SZM3; Q38871 | CPK6; CPK26; CPK5 | 3;2;2 | 7.8836 |
| Q8LDW9 | XTH9 | 4 | 16.761 |
| Q9LSP8 | CMTA6 | 2 | 12.327 |
| Q93VB2 | ATG18A | 6 | 12.213 |
| Q4FE47 | XBAT35 | 1 | 8.2287 |
| O22775; Q9LZJ3 | XXT2; XXT1 | 2;1 | 6.7321 |
| Q9LT17 | BBR | 3 | 6.4213 |
| Q9C519 | WRKY6 | 2 | 6.2971 |
| Q9FKW4 | CPK28 | 3 | 5.0017 |
| Q9FJP6 | PUB38 | 1 | 4.1563 |
| Q9FY48 | KEG | 2 | 2.8538 |
| Q8RXX9 | ATL6 | 1 | 2.3142 |
| O49445 | LECRK72 | 1 | 1.9599 |
| Q9LHP2; Q9SEU4 | SCL30A; SCL33 | 1;1 | 1.7608 |
| Q9ZW18; Q9ZW19 | SAG13; SDR | 1;1 | 1.7468 |
| Q9FFP9 | SRK2H | 7 | 1.6501 |


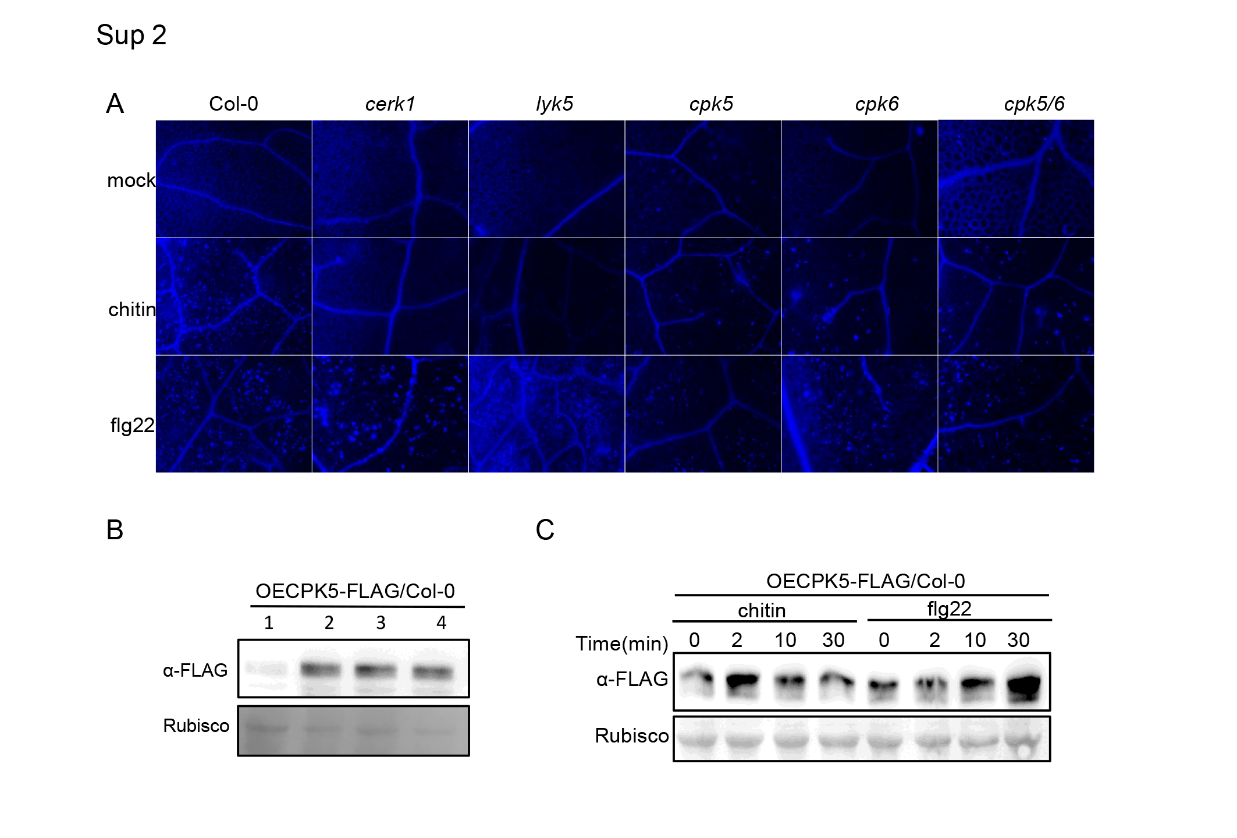


Supplimentary Figure 1. Callose deposition and immunoblot as described below.

1. Callose deposition in Col-0*, Atcerk1-2, Atlyk5-2, Atcpk5, Atcpk6, Atcpk5/6*. For the experiment, leaves were separated from 4-week-old Arabidopsis, and infiltered with chitin (25ug/ml), flg22(500nM) or water for 24 hours. The experiment repeated for twice and shown similar results.
2. Identification protein level of AtCPK5 in each transgenic line. 4-week-old Arabidopsis leaves were separated and perform immunoblot with α-FLAG antibody.
3. AtCPK5 was responsive to chitin. 10-day-old Arabidopsis seedings were treated with chitin (25ug/ml) or flg22(500nM) and harvested at the indicated time for immunoblot analysis with α-FLAG antibody. The experiment was repeated for three times and got similar results.


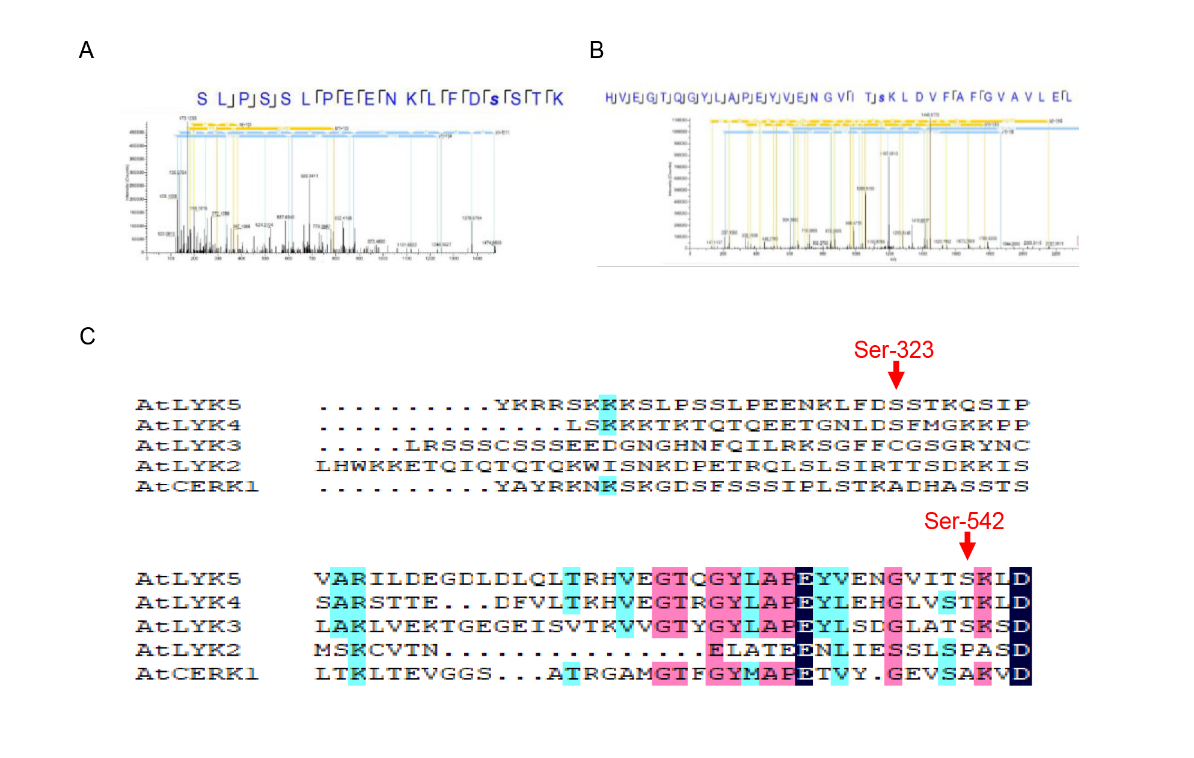


Supplimentary Figure 2. AtLYK5 phospho-site identification and LYKs families protein alignment.

1. and (B) Identified phosphor-sites of AtLYK5-CD at Ser-323 and Ser-542 after kinase reaction in vitro.

(C) Protein sequence alignment of LYKs families in *Arabidopsis.*


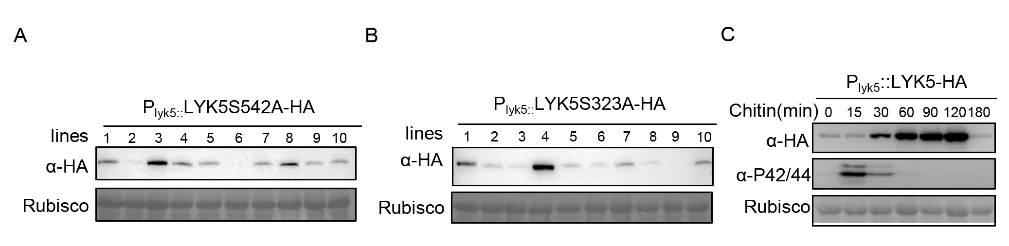


Supplimentary Figure 3. Protein levels of LYK5 phospho-site mutations transgenic lines using immunoblot.

(A) and (B) Four-week -old Arabidopsis leaves were separated from positive transgenic seedlings and performed immunoblot to detect the LYK5 expression using the α-HA antibody. (C) Ten-day-old Arabidopsis seedlings expressing LYK5-HA in *lyk5-2* mutant under its native promoter were treated by chitin and subjected to analyzed LYK5-HA protein level and MAPK cascade activation using the α-HA and α-P42/44 antibody respectively.

Primer list

| qRT-Actin2-F | TCCCTCAGCACATTCCAGCAGAT |
| --- | --- |
| qRT-Actin2-R | AACGATTCCTGGACCTGCCTCATC |
| qRT-NHL10-F | TTCCTGTCCGTAACCCAAAC |
| qRT-NHL10-R | CCCTCGTAGTAGGCATGAGC |
| qRT-WRKY33F | GGTCACAACAATCCGGAAGA |
| qRT-WRKY33R | GGAGAGACAAGAGAAGGAGAGA |
| qRT-CERK1-F | CAAATCAAGAGATGGTGTTGGTGC |
| qRT-CERK1-R | CACCACCCAAACCTCCACT |
| CPK5-LP | ACCGTGACCAGCTAAATGATG |
| CPK5-RP | GAGGAAACAGCGGAGAGAG |
| LB3(cpk5) | CTGAATTTCATAACCAATCTCGATACAC |
| CPK6-LP | CTCGCAACTAACGCTTACCTG |
| CPK6-RP | CTCCATTTCATCGTCTTCTCG |
| LB1.3（cpk6） | ATTTTGCCGATTTCGGAAC |
| MBP-CPK5-F | GAGGGAAGGATTTCAGAATTCATGGGCAATTCTTGCCGT |
| MBP-CPK5-R | AAGCTTGCCTGCAGGTCGACCTACGCGTCTCTCATGCTAATG |
| MBP-CERK1-KD-F | GAGGGAAGGATTTCAGAATTCTATGCTTACCGGAAGAATAAGTCG |
| MBP-CERK1-KD-R | CAAGCTTGCCTGCAGGTCGACCTACCGGCCGGACATAAGAC |
| GST-LYK5-KD-F | GGTCGTGGGATCCCCGAATTCTACAAACGAAGGTCTAAGAAGAAGT |
| GST-LYK5-KD-R | CTCGAGTCGACCCGGGAATTCCTATGGGTCGGCGCG |
| MBP-C2X-CPK6-F | GAGGGAAGGATTTCAGAATTCATGGGCAATTCATGTCGTG |
| MBP-C2X-CPK6-R | CAAGCTTGCCTGCAGGTCGACCTACACATCTCTCATGCTGATGTTTA |
| GST-LYK4-CD-F | GGTCGTGGGATCCCCGAATTCAAGAAGAAAACGAAAACGCAAAC |
| GST-LYK4-CD-R | CTCGAGTCGACCCGGGAATTCTTAGTACGACGATTCTTCCCAG |
| LYK5S323A-F | CAAGCTCTTTGATGCATCAACCAAAC |
| LYK5S323A-R | GTTTGGTTGATGCATCAAAGAGCTTG |
| LYK5S542A-F | TGGAGTCATTACTGCGAAACTAGACG |
| LYK5S542A-R | CGTCTAGTTTCGCAGTAATGACTCCA |
| CERK1D441V-F | TTTATGTCCATAGGGTCATTAAATCTGCCAA |
| CERK1D441V-R | TTGGCAGATTTAATGACCCTATGGACATAAA |
| CPK5D221A-F | TGTGATGCATAGAGCCTTGAAGCCTGAG |
| CPK5D221A-R | CTCAGGCTTCAAGGCTCTATGCATCACA |
| CPK6D209A-F | GTGTTATGCATAGAGCTTTAAAGCCTGAGAAT |
| CPK6D209A-R | ATTCTCAGGCTTTAAAGCTCTATGCATAACAC |
| pENTR-D/TOPO-CPK5-F | GCCCCCTTCGACTCTATGGGCAATTCTTGCCGT |
| pENTR-D/TOPO-CPK5-R | GGCGCGCCCGACCAT CGCGTCTCTCATGCTAATGT |
